# Interactions, Structure and Properties of PLA/lignin/PBAT Hybrid Blends

**DOI:** 10.3390/polym15153237

**Published:** 2023-07-29

**Authors:** Emese Pregi, Imre Romsics, Róbert Várdai, Béla Pukánszky

**Affiliations:** 1 Laboratory of Plastics and Rubber Technology, Department of Physical Chemistry and Materials Science, Faculty of Chemical Technology and Biotechnology, Budapest University of Technology and Economics, Műegyetem rkp. 3, H-1111 Budapest, Hungarypukanszky.bela@vbk.bme.hu (B.P.); 2 Institute of Materials and Environmental Chemistry, Research Centre for Natural Sciences, Magyar Tudósok Körútja 2, H-1117 Budapest, Hungary

**Keywords:** hybrid blends, interactions, miscibility, thermal analysis, tensile testing, dispersed morphology, interpenetrating network, structure–property correlations

## Abstract

Poly(butylene adipate-co-terephthalate) (PBAT) was added to poly(lactic acid) (PLA)/lignin blends to decrease the considerable stiffness and brittleness of the blends. Two- and three-component blends were prepared in a wide composition range through homogenization in an internal mixer followed by compression molding. Interactions among the components were estimated by comparing the solubility parameters of the materials used and through thermal analysis. Mechanical properties were characterized by tensile testing. The structure of the blends was studied using scanning electron (SEM) and digital optical (DOM) microscopy. The results showed that the interactions between PBAT and lignin are somewhat stronger than those between PLA and the other two components. The maleic anhydride grafted PLA added as a coupling agent proved completely ineffective; it does not modify the interactions. The structural analysis confirmed the immiscibility of the components; the structure of the blends was heterogeneous at each composition. A dispersed structure formed when the concentration of one of the components was small, while, depending on lignin content, an interpenetrating network-like structure developed and phase inversion took place in the range of 30–60 vol% PBAT content. Lignin was located mainly in the PBAT phase. Properties were determined by the relative amount of PBAT and PLA; the addition of lignin deteriorated properties, mainly the deformability of the blends. Other means, such as reactive processing, must be used to improve compatibility and blend properties. The results contribute considerably to a better understanding of structure–property correlations in lignin-based hybrid blends.

## 1. Introduction

The production and use of plastics are increasing continuously and eventually result in immense environmental pollution [1]. One of the possibilities to decrease plastic waste is the use of materials from natural resources [2]. The application of natural and synthetic biopolymers has increased exponentially recently. Poly(lactic acid) (PLA) is the thermoplastic biopolymer produced in the largest quantity at present [3]. It is often used for 3D printing [4], a promising additive manufacturing process [5,6]. Despite having an excellent property combination and its main advantage of being produced from raw materials originating in nature, PLA also has some deficiencies. It is relatively brittle [7,8] and sensitive to water during processing [7,9], its physical aging is fast because of its relatively low glass transition temperature [10,11], it crystallizes rather slowly [11,12] and it is quite expensive compared to commodity polymers [2]. Nevertheless, PLA is the most researched and widely used polymer these days, and it is modified in many ways to improve its property profile, including blending [13,14,15,16], fiber reinforcement [17,18,19,20,21], plasticization [22,23,24], etc.

Lignin is the second most abundant natural polymer produced by nature [25]. Lignin forms as a by-product in several industrial processes, including the production of cellulose and bioethanol, and it is cheap; thus, its use would offer several advantages [26]. Recently, many attempts have been made to apply lignin as a component of plastics to decrease the price, possibly improve the mechanical properties and decrease the carbon footprint of the final product. Blends have been prepared from lignin and a wide variety of thermoplastic polymers [26,27,28], it has been used as a reactive component in phenolic resins [28,29,30] and in polyurethanes [31,32,33], polyolefin polymers have been stabilized with it [34,35,36], etc. The combination of PLA and lignin seems an obvious approach since lignin is a biopolymer available in large quantities at a relatively low price. Therefore, many research groups attempted to prepare blends from the two components, but their blending resulted in materials with poor properties. Adding lignin up to 70 vol% to PLA increased the stiffness but decreased the tensile strength and elongation-at-break of the blends [37]. The large stiffness but small strength, deformability and impact resistance of PLA/lignin blends are hindering their future practical application [37,38,39,40].

The fast physical ageing of PLA results in an increase in its stiffness and strength but also in a decrease in its deformability [10,41]. The elongation-at-break of a PLA specimen is larger than 100% after its production but decreases to below 10% in a few weeks [10]. The ductility of PLA can be increased through plasticization, but the approach results in a drastic decrease in stiffness [22,23,24]. The use of impact modifiers seems to be a better solution to increase ductility since the modulus of the material does not decrease to the same extent as in the case of plasticization [22]. Poly(butylene adipate-co-terephthalate) (PBAT) is a biopolymer that is often used to improve the properties of PLA, mostly to increase its impact resistance [16,42,43,44]. Depending on its composition, the copolymer is compostable, thus preserving the biodegradability of its blends [45,46]. Adding PBAT to a PLA/lignin blend seems to be an obvious way to improve the properties of two-component blends.

Hybridization is thought to be an approach that solves all problems related to the structure and properties of heterogeneous blends and composites. All kinds of hybrids are prepared from a very wide variety of materials, including the combination of three polymers [47,48,49,50,51,52,53], two polymers and a filler [54,55,56,57] or fiber [58,59,60], or a polymer matrix and two reinforcements [61,62,63,64,65], and significant improvement in properties as well as the synergistic effect of the components are claimed in the majority of cases [63,64,65]. However, hybridization raises many questions, and without addressing them, the claimed property improvement might not occur. Moreover, proper efforts are rarely made to prove the existence of synergy [61]. Interactions determine the mutual miscibility of the polymer components, the adhesion of the phases to each other and, thus, structure and properties [26]. In the case of three-component materials, the components can be dispersed independently of each other in a matrix [66,67,68,69], or one component might be encapsulated by one of the polymers [68,69,70,71]. The structure determines properties which can vary in a wide range depending upon the actual morphology of the material [68,69].

Because of the limited practical application of PLA/lignin blends caused by their poor mechanical properties, it is worth studying the effect of hybridization on these blends. In accordance with the considerations presented above, the goal of this study was to explore the possibility of improving the mechanical properties, and especially the brittleness, of PLA/lignin blends by adding PBAT as a third component. Two- and three-component blends were prepared from PLA, Kraft lignin and PBAT in a wide composition range. Attempts were made to estimate the interactions developing among the components, and the ensuing structure was characterized through the analysis of the composition dependence of properties and through microscopy. Interactions were also modified by adding a functionalized, maleated PLA polymer. Correlations between the structure and properties of the blends were analyzed in detail, and aspects of the possible practical application of the blends were considered and are also mentioned in the final section of this paper.

## 2. Materials and Methods

### 2.1. Materials

The PLA used as matrix in the study was the Ingeo 4032D grade supplied by NatureWorks (NatureWorks LLC, Minnetonka, MN, USA). The polymer (<2% D isomer) had a melt flow rate (MFR) of 7 g/10 min at 210 °C, 2.16 kg load, and a density of 1.24 g/cm^3^. The PBAT used was the Ecoflex F Blend C1200 grade (BASF SE, Ludwigshafen, Germany), which is a biodegradable, statistical, aliphatic–aromatic copolyester. The PBAT had a density of 1.25 g/cm^3^ and a melt flow rate (MFR) of 2.7–4.9 g/10 min at 190 °C, 2.16 kg load. The Indulin AT Kraft lignin used to prepare the blends was supplied by Ingevity (Ingevity Corp., North Charleston, SC, USA). Indulin AT is a purified form of Kraft pine lignin and is completely free of all hemicellulosic materials. Its C_9_ formula is C_9_H_8.53_O_1.85_(OCH_3_)_1.02_N_0.078_S_0.080_ [72]. The Kraft lignin used is an industrial lignin grade, and therefore it has a relatively low molecular weight (M_n_ = 1100–1300 g/mol) [72,73]. Its ash content is around 3% [72,74], it has a density of 1.3 g/cm^3^ and it contains about 1.5% of various sugars [74]. The concentration of functional groups capable of forming hydrogen bonds was determined through ^31^P NMR [72]. The Kraft lignin contains 2.59 mmol/g aliphatic hydroxyl, 4.00 mmol/g phenolic hydroxyl and 0.20 mmol/g carboxyl groups. The average diameter of the lignin particles before blend preparation was 77 µm.

The maleic anhydride grafted PLA coupling agent (MAPLA) was prepared in our laboratory. Its production technology was described earlier in detail [18]. The PLA used in the grafting reaction was the Ingeo 3251D grade, also obtained from NatureWorks (NatureWorks LLC, Minnetonka, MN, USA) (MFR = 35 g/10 min at 190 °C and 2.16 kg load). A Brabender LabStation (Brabender GmbH, Duisburg, Germany) single-screw extruder was used for reactive extrusion. The temperature profile was 175–180–185–190 °C, and the screw speed was 12 rpm. The reaction mixture contained 2 wt% maleic anhydride and 2 wt% Luperox 101 peroxide as the initiator. The MAPLA was characterized through NMR (Varian NMR System, Agilent Technologies, Inc., Santa Clara, CA, USA). However, it was not purified; it was used as produced in the reactive extrusion.

The composition of the blends changed in a wide range. The lignin content of the PLA/lignin blends varied between 0 and 50 vol% and that of the PBAT/lignin blends between 0 and 70 vol%, respectively, in 10 vol% increments in both cases. Two-component blends were prepared from PLA and PBAT in the entire composition range; composition changed in 10 vol% steps in this case, too. Hybrid blends contained 10, 20 and 30 vol% lignin, and their PBAT content changed between 0 and 80 vol%, respectively, in 10 vol% steps. MAPLA was added to improve the interaction between PLA and the other two components, i.e., PLA/lignin, PLA/PBAT and PLA/lignin/PBAT blends, in 10 wt% calculated for the amount of the dispersed component.

### 2.2. Sample Preparation

Before processing, PLA, lignin and the coupling agent were dried in a vacuum oven (Memmert VO500, Memmert GmbH, Schwabach, Germany) at 105 °C and 150 mbar pressure for 4 h to eliminate the moisture absorbed during standing. PBAT was dried at 80 °C for 4 h in an air-circulating oven (Memmert UF450, Memmert GmbH, Schwabach, Germany). The components were homogenized in a Brabender W 50 EHT internal mixer (Brabender GmbH, Duisburg, Germany) at 190 °C set temperature, 47 cm^3^ charge volume and 50 rpm. The materials were added into the mixer in the order of PLA, MAPLA, PBAT and lignin. Mixing time was 10 min after the addition of the last component. After homogenization, plates of 1 mm thickness were compression-molded at 190 °C in 6 min using a Fontijne SRA 100 machine (Fontijne Presses b.v., Vlaardingen, The Netherlands). Tensile bars were machined from the plates for further testing after storing them for one week at room temperature. The sample preparation process is visualized in Figure S1 and the tensile test specimen dimensions (Figure S2 and Table S1) can be found in the Supplementary Materials.

### 2.3. Characterization and Measurements

To determine relaxation transitions and the glass transition temperature of the polymer components, dynamic mechanical thermal analysis (DMTA) was carried out on specimens with 50 × 5 × 1 mm dimensions between −150 °C and the failure of the sample at 1 Hz frequency, 10 μm deformation and a 2 °C/min heating rate. The measurements were performed using a PerkinElmer Diamond DMA (Perkin Elmer Inc., Waltham, MA, USA). Relaxation transitions, as well as the melting and crystallization of the components in the blends, were studied through differential scanning calorimetry (DSC) using a Perkin Elmer DSC 7 (Perkin Elmer, Inc., Norwalk, CT, USA) apparatus. The measurements were carried out in two heating runs and one cooling run between −50 °C and 200 °C with heating and cooling rates of 10 °C/min. The weight of the samples was 3–5 mg in each case. Mechanical properties were characterized through tensile testing using an Instron 5566 (Instron, Norwood, MA, USA) universal testing machine. The gauge length was 80 mm and the test was performed at 10 mm/min crosshead speed. Five parallel measurements were carried out on each material. The structure of the blends was analyzed through scanning electron microscopy (SEM) using a Jeol JSM 6380 LA apparatus (Jeol Ltd., Tokyo, Japan). Thin slices were cut from the 1 mm thick plates at −80 °C using a Leica EM UC6 microtome (Leica, Microsysteme GmbH, Wien, Austria), and then the lignin was dissolved from the slices by soaking them in a 70:30 mixture of acetone and distilled water for 24 h at ambient temperature. The quality of the slices was checked, and the structure of the materials was also studied through digital optical microscopy (DOM) using a Keyence VHX 5000 (Keyence Co., Osaka, Japan) apparatus.

## 3. Results

The results are presented in several sections. Interactions and the effect of the functionalized PLA on them are considered first based on thermal analysis. The structure of the two- and three-component blends is analyzed next, followed by the presentation of the composition dependence of tensile properties. Structure–property correlations and consequences for practice are considered in the final section of the paper.

### 3.1. Interactions and Miscibility

Interactions always develop between or among the components in multicomponent materials. In the case of blends, these interactions lead to the mutual miscibility of the components. However, when interactions are weak, limited miscibility may occur, leading to the formation of various phases and a complicated heterophase structure. The simplest way to estimate interactions in polymer blends is the comparison of the solubility parameters of the components. Although the approach has many deficiencies, it gives a rough idea about interactions and the possible structure of the blends. Solubility parameters for the components in question were reported in the literature by several groups [26,37,75,76,77,78,79,80,81]. The values for PLA covered the range between 18.7 and 22.7 MPa^1/2^ [37,76,79,80]; it was predicted as 20.5-22.2 MPa^1/2^ [75,77,79,80] for PBAT and located between 23.3 and 27.5 MPa^1/2^ [26,78,81] for Kraft lignin. According to the literature, the most probable values are 19.5, 21.5 and 24.0 MPa^1/2^ for the three components, respectively. The largest value in the literature was found for lignin and the smallest for PLA, in accordance with the expectations. Based on these results, immiscibility and a heterogeneous structure can be predicted for the studied blends, along with the development of stronger interactions between PBAT and lignin than between PLA and lignin.

Another relatively simple way to estimate interactions is thermal analysis. In the case of complete miscibility, the blend has only one glass transition temperature (T_g_), and the lack of miscibility results in transition temperatures corresponding to those of the components [82]. In the present case, the situation is rather complicated; the blend contains at least three components, PLA, lignin and PBAT, and additionally, the two polyesters may also crystalize to various extents.

**Figure 1:**
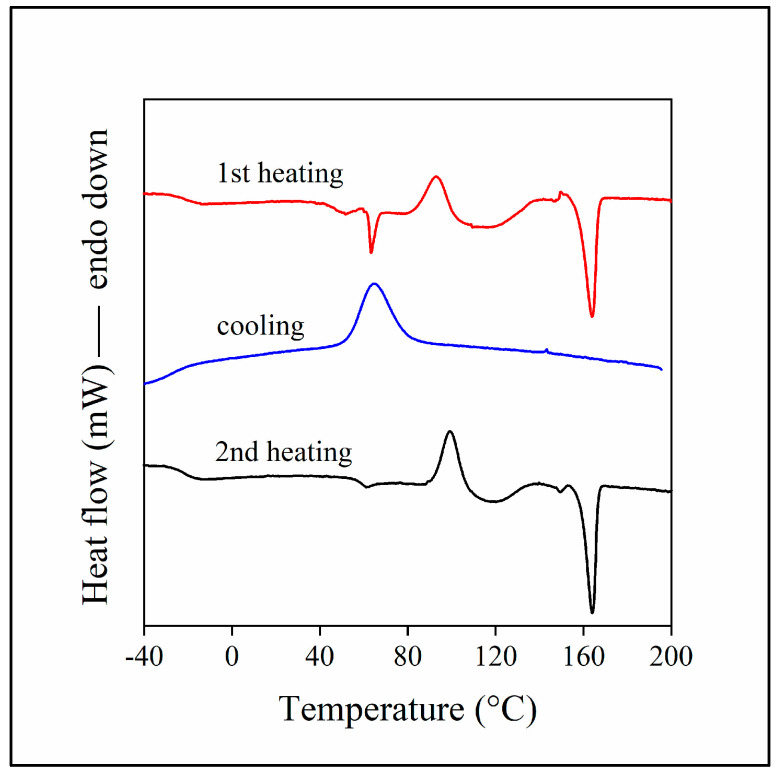
The results of DSC measurements on a PLA/lignin/PBAT blend containing 20 vol% lignin, 60 vol% PBAT and the functionalized PLA. Two heating runs (1st heating: red line, 2nd heating: black line) and a cooling run (blue line).

The complicated situation is demonstrated well by Figure 1, presenting the DSC traces of the PLA/lignin/PBAT blend containing 20 vol% lignin, 60 vol% PBAT and also the compatibilizer (MAPLA). The figure shows the curves recorded in the two heating runs and a cooling run. The glass transition of PBAT appears at sub-zero temperatures, at around −20 °C. This is followed by the glass transition of PLA at around 60 °C, which is quite intensive in the first heating because of the fast cooling during sample preparation. Cold crystallization of PLA occurs subsequently at around 100 °C, followed by the melting of the PBAT component. The crystalline PLA phase melts at around 164 °C. No transition can be detected for the lignin component. This is not surprising since lignin molecules consist of several aromatic rings. Lignin molecules and the forming lignin phase are very stiff; thus, transitions are difficult to detect. However, it is evident from the figure that PLA and PBAT form separate phases in the blend; their mutual miscibility is very limited. A comparison of the DSC traces recorded in the first heating run of the neat polymers and the hybrid blend is presented in Figure S3 in the Supplementary Materials.

**Figure 2:**
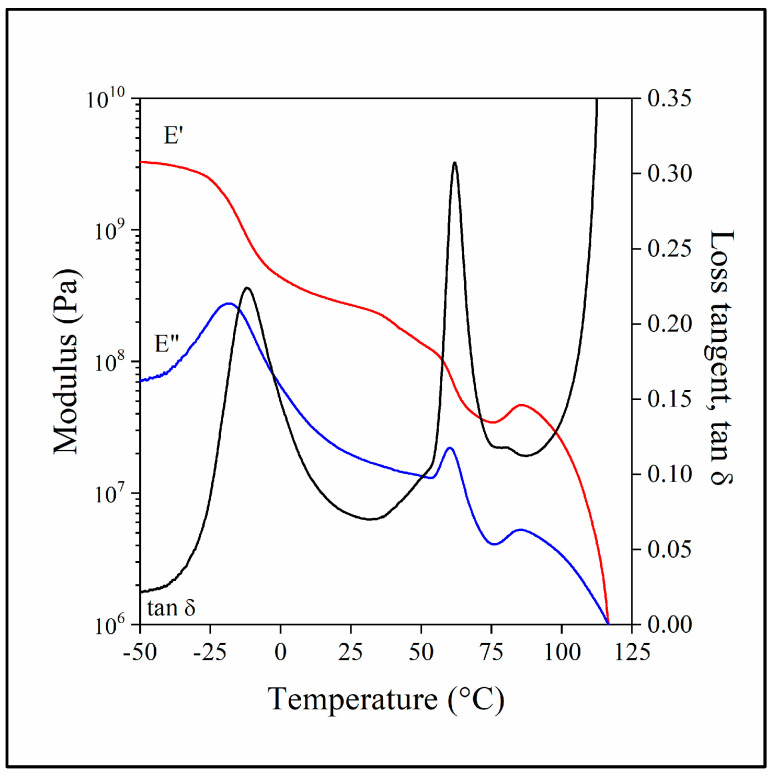
Dynamic mechanical spectra recorded on the PLA/lignin/PBAT blend containing 20 vol% lignin, 60 vol% PBAT and MAPLA; temperature dependence of storage modulus (red line), loss modulus (blue line) and tan δ (black line).

Dynamic mechanical thermal analysis (DMTA) offers similar information on transitions taking place during the heating of the material but with a different emphasis on certain transitions. Accordingly, the spectra are somewhat simpler than the DSC traces shown above. The DMTA spectra of a blend containing 20 vol% lignin, 60 vol% PBAT and MAPLA are presented in Figure 2. The glass transition of the PBAT phase is much more obvious in this figure than on the DSC trace. The glass transition of PLA is also more intense, and it is followed by the modulus increase caused by the cold crystallization of this polymer. DMTA measurements confirmed the conclusion presented above, i.e., PLA and PBAT form two separate phases with separate glass transitions. Both thermal analysis techniques also proved that the two main components, PLA and PBAT, crystallize to some extent, and the crystalline phases form independently. A comparison of the loss tangent values of the neat polymers and the hybrid blend is presented in Figure S4, and the composition dependence of the glass transition temperature of PLA is shown in Figure S5 in the Supplementary Materials.

**Figure 3:**
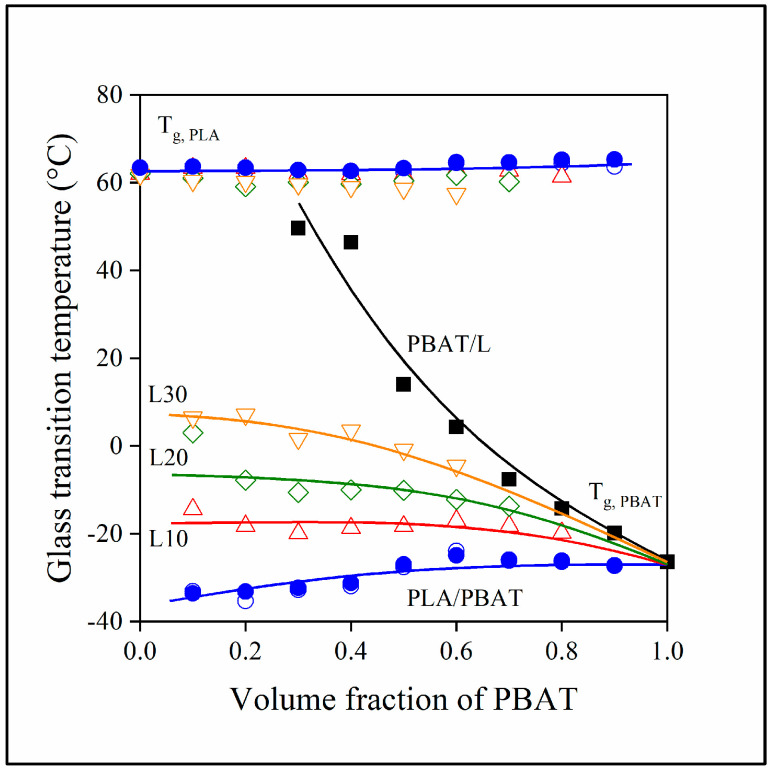
Composition dependence of the glass transition temperature of PLA and PBAT in the two- and three-component blends studied. (Symbols: (●) PLA/PBAT; (■) PBAT/lignin; lignin content in three-component blends: (△) 10, (◇) 20, (▽) 30 vol%. Empty symbols with and full symbols without MAPLA).

Although Figure 1 and Figure 2 prove that the miscibility of the components is limited and that they form separate phases, they do not offer any information about the strength of interactions. Such information might be provided by the analysis of changes in the glass transition temperature of the components as a function of composition. The T_g_ values of the components are plotted against the PBAT content of the blends in Figure 3. The T_g_ of PLA practically does not change significantly with PBAT or with lignin content, and the presence of MAPLA does not influence the transition temperature. Consequently, PLA develops only very weak interactions with other components, and adding the functionalized PLA does not modify interactions either.

On the other hand, the interaction of PBAT and lignin is quite strong, and the glass transition temperature of the former increases strongly with increasing lignin content. The interaction of these two components is also confirmed in the three-component blends; the T_g_ of PBAT increases with increasing PLA content and the slope of the increase is proportional to lignin content. Since the glass transition temperature of lignin cannot be determined with either technique, it is impossible to establish if lignin dissolves in PBAT and to what extent, or whether lignin behaves as a coupling agent between the two polyesters. These questions might be answered using microscopy, through the analysis of structure.

### 3.2. Structure

**Figure 4:**
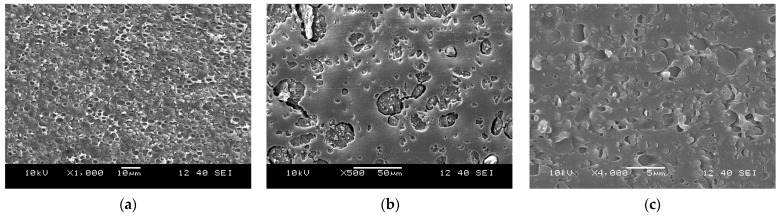
Micrographs recorded for the structure of two-component blends: (**a**) PLA/PBAT, 30 vol% PBAT; (**b**) PLA/lignin, 30 vol% lignin; (**c**) PBAT/lignin, 30 vol% lignin.

In the case of limited miscibility—and thermal analysis clearly proves that in the blends in question, that is the case—dispersed structure forms when the concentration of one of the components is small. This general statement is clearly confirmed by Figure 4, showing the dispersion of one component in another. The SEM micrograph recorded on the fractured surface of a PLA/PBAT blend is presented in Figure 4a. PBAT is dispersed as small, micron-sized particles in the PLA matrix. According to thermal analysis, interactions are not strong between PLA and lignin either, which is confirmed by Figure 4b, showing the dispersion of lignin particles in PLA. The size of the particles is larger than in the previous PLA/PBAT blend, indicating an even larger extent of phase separation in PLA/lignin than in PLA/PBAT blends. Figure 3 indicated strong interaction between PBAT and lignin, and the question even arose whether the two components are completely miscible. Stronger interactions are confirmed by Figure 4c, but miscibility is not. Small lignin particles are dispersed in the PBAT matrix at 30 vol% lignin content. The SEM study of the structure of two-component blends confirmed that all components are immiscible and form separate phases upon blending. Figure S6 also proves that the coupling agent does not influence the microstructure of the blends.

**Figure 5:**
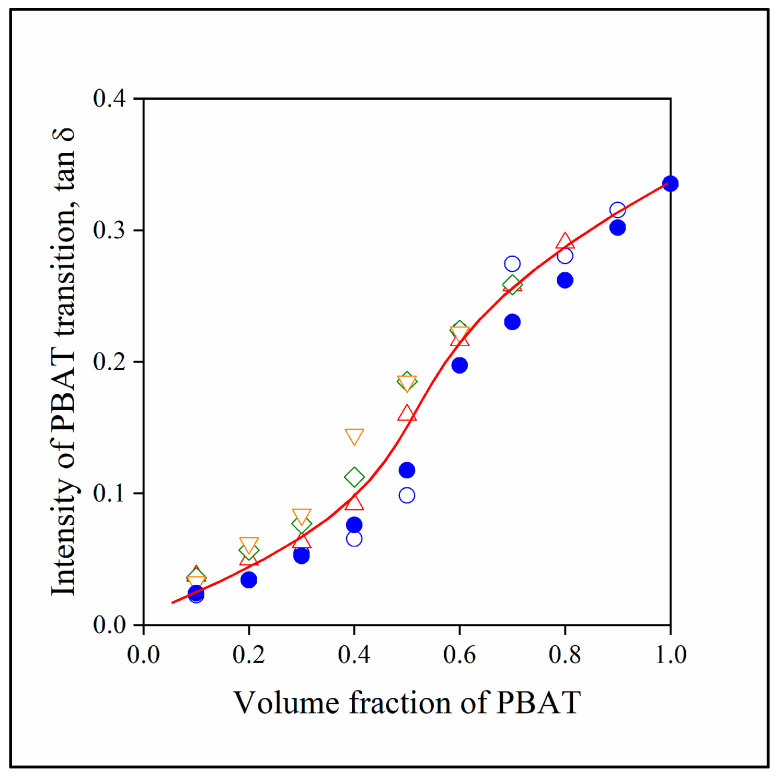
Composition dependence of the intensity of the glass transition (tan δ) of PBAT in the two- and three-component blends studied. (Symbols: (●) PLA/PBAT; lignin content in three-component blends: (△) 10, (◇) 20, (▽) 30 vol%. Empty symbols with and full symbols without MAPLA).

In the blends of two immiscible polymers, phase inversion must take place at an intermediate composition. In the PLA/PBAT blends, this occurs in the range of 50–60% PBAT content, as shown by Figure 5, presenting the composition dependence of the intensity of loss tangent of the PBAT glass transition. The sudden increase in the intensity of tan δ in the range mentioned clearly proves phase inversion, which is further confirmed by the composition dependence of other properties like elongation-at-break (see below).

**Figure 6:**
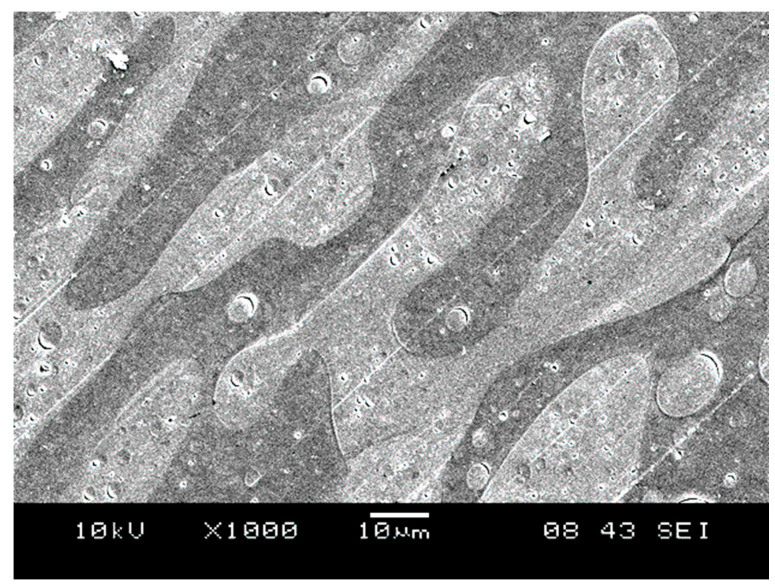
Interpenetrating network-like structure in a PLA/PBAT blend of 50/50 vol% composition. The blend also contained MAPLA.

Further proof for phase inversion is supplied by the SEM micrograph presented in Figure 6, recorded for a PLA/PBAT blend of 50/50% composition. The blend also contained the compatibilizer, but its presence did not influence the structure. Clearly, an interpenetrating network-like structure forms in this blend, which is usual in the composition range of phase inversion. The relatively narrow concentration range of phase inversion further indicates the quite poor interaction and limited miscibility of the two polymers.

**Figure 7:**
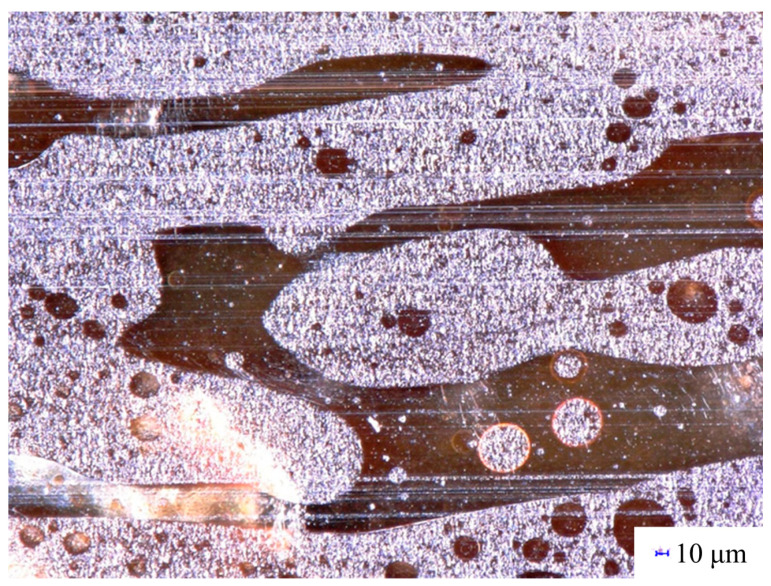
DOM micrograph showing the location of lignin in a PLA/lignin/PBAT blend at 30 vol% lignin and 30 vol% PBAT content.

The only remaining question is the location of lignin particles in the three-component blends. As we saw earlier, all components are immiscible with each other. Thus, lignin must be located in one of the two phases or at the interphase of the two polyester components. Because of the stronger interactions between PBAT and lignin, the latter is located mainly in the PBAT phase, as shown in Figure 7. The DOM micrograph clearly confirms the formation of an interpenetrating network-like structure already at around 30 vol% PBAT content and shows that the majority of lignin is located within the PBAT phase. The embedding of lignin into PBAT may have extended the range of interpenetrating network-structure compared to the two-component PLA/PBAT blend. However, we must note here that drawing conclusions from a few SEM or DOM micrographs might be misleading, and such results must be treated carefully.

### 3.3. Properties

**Figure 8:**
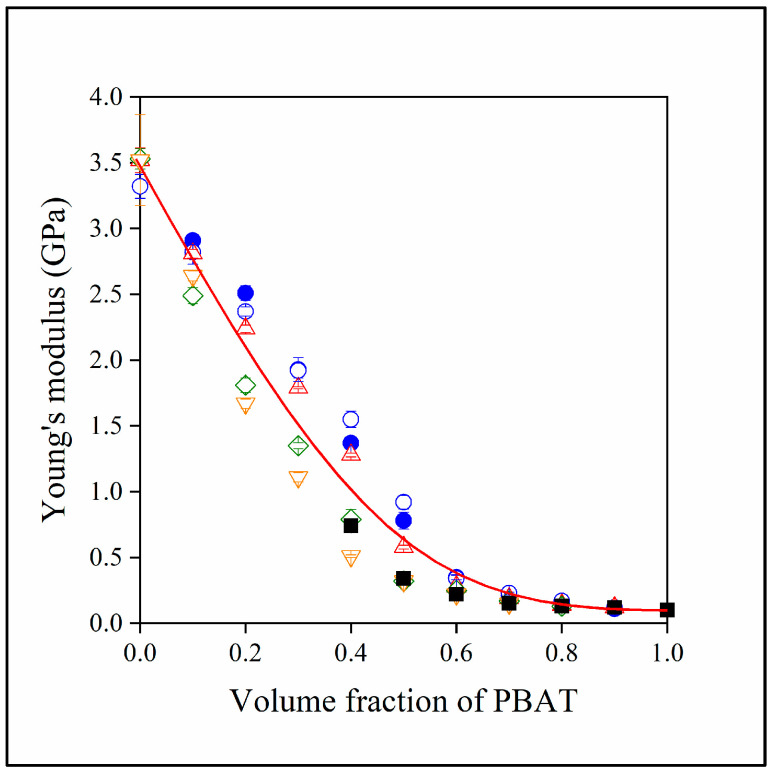
Young’s modulus of the two- and three-component blends studied plotted against their PBAT content. (Symbols: (●) PLA/PBAT; (■) PBAT/lignin; lignin content in three-component blends: (△) 10, (◇) 20, (▽) 30 vol%. Empty symbols with and full symbols without MAPLA).

The major disadvantages of polymer/lignin blends have always been heterogeneity and brittleness. The strong interactions among lignin molecules lead to phase separation, and the stiff lignin particles decrease deformability. The goal of preparing three-component hybrid blends was to improve this latter property through the beneficial effect of PBAT. The stiffness of two- and three-component blends is presented in Figure 8. The moduli of the two components, PLA and PBAT, differ considerably from each other. Consequently, the modulus of the blends changes continuously between the two values as PBAT content increases. Structure influences stiffness only slightly, but at least one conclusion can be drawn about structure from the results. Close scrutiny shows that instead of increasing, stiffness decreases with increasing lignin content. Lignin particles are very stiff and increase the modulus of practically all polymers containing them (see black squares). The decrease in stiffness with increasing lignin content is the result of the embedding of lignin into PBAT, which confirms our conclusion about structure as discussed previously.

**Figure 9:**
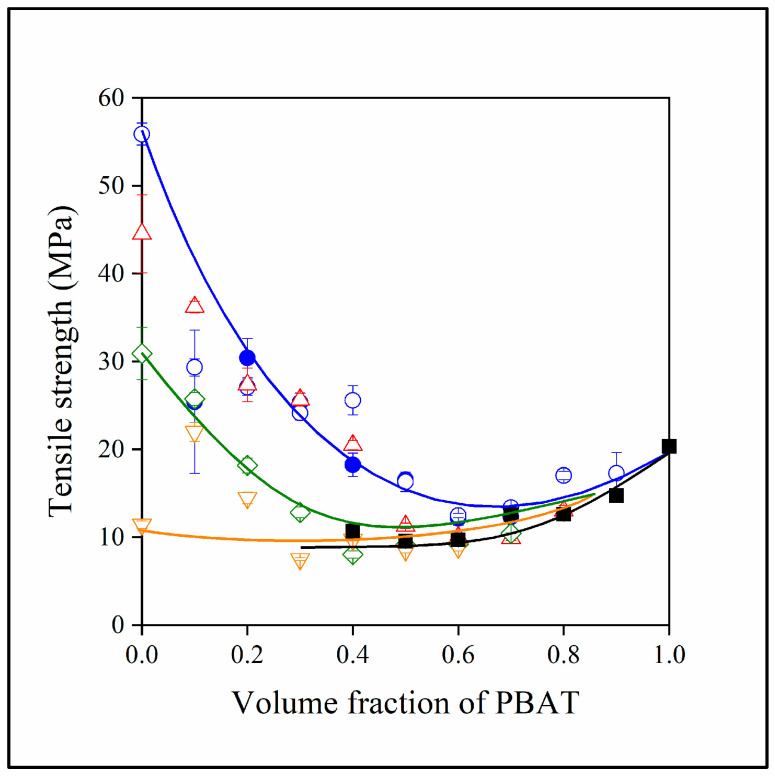
Effect of PBAT content on the tensile strength of the two- and three-component blends investigated. (Symbols: (●) PLA/PBAT; (■) PBAT/lignin; lignin content in three-component blends: (△) 10, (◇) 20, (▽) 30 vol%. Empty symbols with and full symbols without MAPLA).

Properties measured at larger deformations, such as strength and elongation-at-break, reflect the effect of interactions and structure better than stiffness. Because of incompatibility and its smaller load-bearing capacity, PBAT decreases the tensile strength of PLA quite substantially (Figure 9). Because of immiscibility, lignin decreases the strength of PBAT as well (see black squares ■). The combined effect of immiscibility and the embedding of lignin into the PBAT phase results in the drastic decrease in tensile strength in the case of the three-component blends. Although this result is in complete agreement with thermal analysis and the conclusion drawn from the study of structure, the outcome is rather disadvantageous; the addition of PBAT to PLA/lignin blends does not improve properties to the extent hoped for.

**Figure 10:**
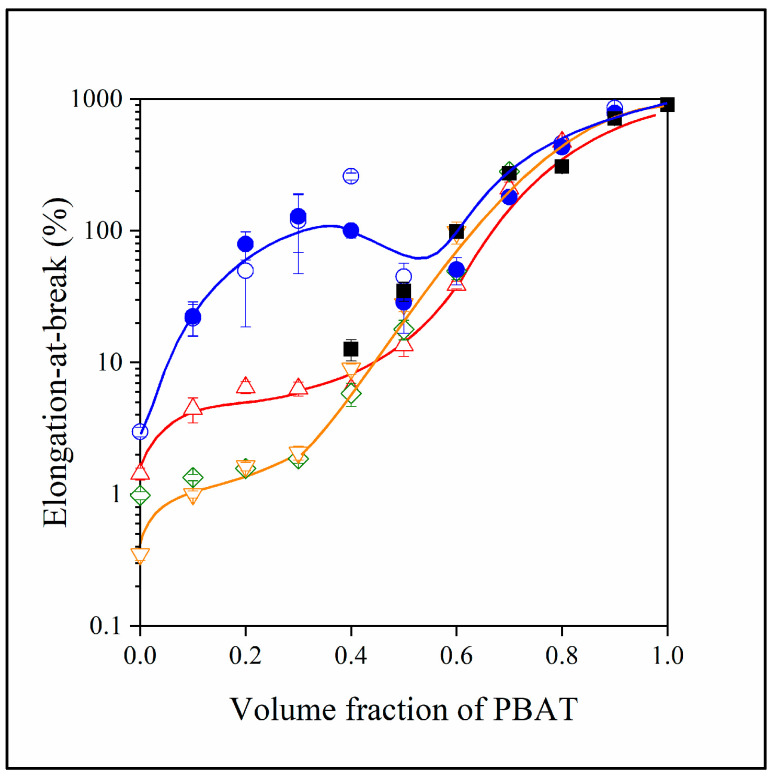
Composition dependence of the elongation-at-break of two- and three-component blends at various combinations of the components. (Symbols: (●) PLA/PBAT; (■) PBAT/lignin; lignin content in three-component blends: (△) 10, (◇) 20, (▽) 30 vol%. Empty symbols with and full symbols without MAPLA).

As mentioned above, the main idea of adding PBAT to PLA/lignin blends was to increase deformability. The elongation-at-break of the various blends is plotted against PBAT content in Figure 10. Because of the significant difference in the elongation-at-break of PLA and PBAT, the results can be presented only on a logarithmic scale. The addition of PBAT increases the deformability of PLA indeed, but lignin decreases it considerably for all combinations of the polymers in both PBAT (■) and the three-component PLA/lignin/PBAT blends. Phase inversion is shown by the change in elongation at the intermediate composition range, and the inefficiency of MAPLA to mediate interactions is confirmed again, just like in the case of all other properties. The modulus, tensile strength and elongation-at-break values are summarized in Tables S2–S4 in the Supplementary Materials.

## 4. Discussion

Preliminary considerations, as well as thermal analysis, proved that the interactions among the components of PLA/lignin/PBAT blends are weak; the components are immiscible in each other. Immiscibility leads to a heterogeneous structure, dispersed particles of the minor component at the extremes of the composition range and an interpenetrating network-like structure in its middle. Due to thermodynamic reasons, lignin is located mainly in the PBAT phase. The structure of the blends was confirmed using microscopy, and the composition dependence of properties also corroborated the conclusions.

**Figure 11:**
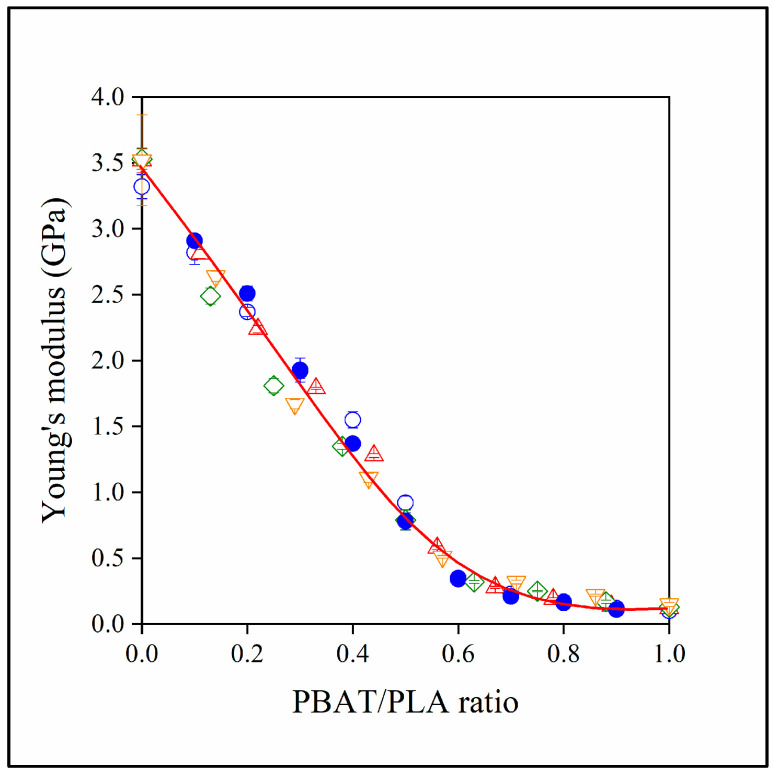
Effect of the PBAT/PLA ratio on the stiffness (Young’s modulus) of the studied two- and three-component blends. (Symbols: (●) PLA/PBAT; lignin content in three-component blends: (△) 10, (◇) 20, (▽) 30 vol%. Empty symbols with and full symbols without MAPLA).

The fact that lignin is located in the PBAT phase leads to the strange result that its presence influences properties only to a limited extent. According to the results, properties are determined by the relative amount of PBAT/PLA in the blends. Adding lignin to the two-component blend changes the composition, the relative ratio of the two polyesters and, thus, also the composition dependence of properties. However, if we plot properties against the PBAT/PLA ratio, we obtain a unique correlation, as shown in Figure 11 for modulus.

**Figure 12:**
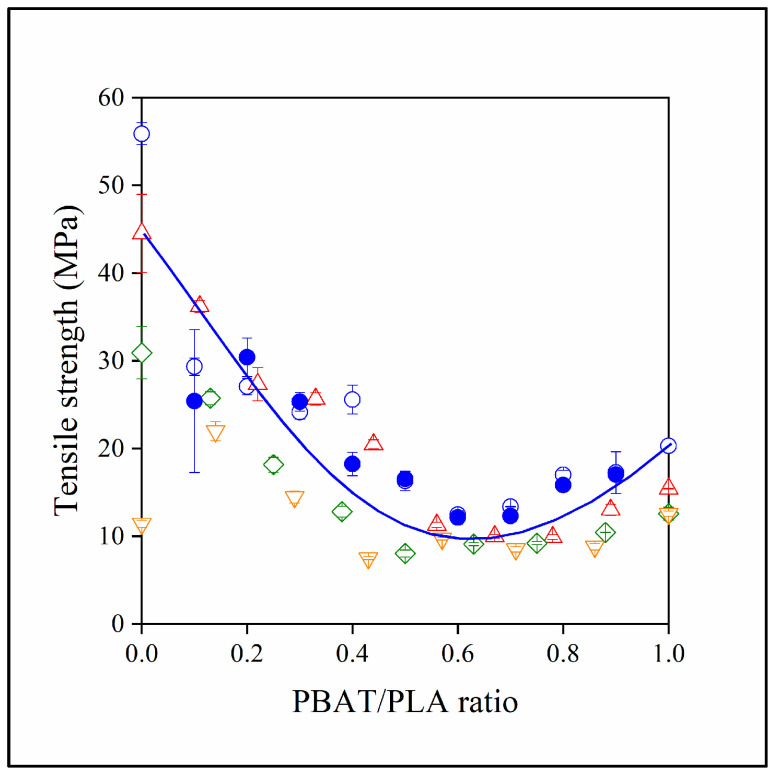
Dependence of the tensile strength of the two- and three-component blends investigated on the ratio of PBAT and PLA. (Symbols: (●) PLA/PBAT; lignin content in three-component blends: (△) 10, (◇) 20, (▽) 30 vol%. Empty symbols with and full symbols without MAPLA).

The correlation is very close with hardly any deviation. Even the embedding of lignin into the PBAT phase does not result in the deviation of the points from the general correlation. Larger scattering of the points can be observed in the case of the tensile strength (see Figure 12) and embedding has a more significant influence than in the previous case. Nevertheless, the dominating factor is clearly the relative amount of PBAT and PLA here too. This is clearly shown and emphasized by the strength of the PLA/lignin blends. Lignin decreases the strength of PLA drastically, from close to 60 down to 10 MPa, while the effect is much more moderate in the presence of PBAT.

All results indicate that the addition of PBAT does not yield the desired results, and the properties of PLA/lignin blends do not improve upon the addition of PBAT. The functionalized, maleated PLA does not act as a compatibilizer or coupling agent, and thus it does not improve interactions among the phases. Properties remain mediocre at all compositions, and apart from the large PBAT content, the deformability of the blends is very limited. The brittleness of the blends seriously limits their practical application. Other means, e.g., reactive processing, must be used to improve the properties of PLA/lignin and PLA/lignin/PBAT blends.

## 5. Conclusions

PBAT was added to PLA/lignin blends in order to improve their mechanical properties, and especially to decrease brittleness. Theoretical considerations and thermal analysis showed that the interactions among all components are weak; they are immiscible with each other. The interactions between PBAT and lignin are somewhat stronger than those between PLA and the other two components. The functional PLA added as a coupling agent proved completely ineffective. Structural analysis confirmed the immiscibility of the components; a heterogeneous structure formed at all compositions. Minor components were dispersed in the matrix of the major one at the extremes of the composition range, while an interpenetrating network-like structure formed, and phase inversion took place at intermediate compositions. The width of the IPN-like structure is relatively narrow, proving the formation of weak interactions among the components again. Properties are determined by the relative amount of PBAT and PLA, and the addition of lignin deteriorates properties, mainly the deformability of the blends. The expected improvement in properties has not been achieved; other means, such as reactive processing must be used to improve compatibility and blend properties. However, these results contribute to a better understanding of the structure–property correlations in lignin-based hybrid blends and help in the design of multicomponent polymer systems.

## Data Availability

The raw/processed data required to reproduce these findings cannot be shared at this time as the data also form part of an ongoing study.

## Supplementary Materials

The following supporting information can be downloaded at: https://www.mdpi.com/article/10.3390/polym15153237/s1. Figure S1: The preparation process of the samples; Figure S2: Shape and dimensions of tensile test specimens; Figure S3: The result of a DSC measurement on a neat PLA, neat PBAT and PLA/lignin/PBAT blend containing 20 vol% lignin, 60 vol% PBAT and the functionalized PLA. First heating run; Figure S4: Dynamic mechanical spectra recorded on the neat PLA, neat PBAT and PLA/lignin/PBAT blend containing 20 vol% lignin, 60 vol% PBAT and MAPLA; temperature dependence of tan δ; Figure S5: Composition dependence of the glass transition temperature of PLA in the two- and three-component blends studied. Symbols: (●) PLA/PBAT; (■) PBAT/lignin; lignin content in three-component blends: (△) 10, (◇) 20, (▽) 30 vol%. Empty symbols with and full symbols without MAPLA; Figure S6: Micrographs recorded on the structure of two-component PLA/PBAT blends containing 30 vol% of PBAT: (a) with MAPLA; (b) without MAPLA; Table S1: Dimensions of tensile test specimens; Table S2: Mechanical properties of two-component blends; Table S3: Mechanical properties of two-component blends containing MAPLA; Table S4: Mechanical properties of three-component hybrid blends containing MAPLA.
