# Supplementary material for: Interactions, Structure and Properties of PLA/lignin/PBAT Hybrid Blends
Source: Polymers (Basel). 2023 Jul 29;15(15):3237. doi: 10.3390/polym15153237 (PMC10422597; doi:10.3390/polym15153237)
Supplement: Supplementary file 1 [file polymers-15-03237-s001.zip › polymers-2467161-supplementary.pdf]

Supplementary Information on

INTERACTIONS, STRUCTURE AND PROPERTIES IN PLA/LIGNIN/PBAT HYBRID BLENDS

Emese Pregi [1,2,*], Imre Romsics [1,2], Róbert Várdai [1,2] and Béla Pukánszky [1,2]

[1] Laboratory of Plastics and Rubber Technology, Department of Physical Chemistry and Materials Science, Faculty of Chemical Technology and Biotechnology, Budapest University of Technology and Economics, Műegyetem rkp. 3., H-1111 Budapest, Hungary
[2] Institute of Materials and Environmental Chemistry, Research Centre for Natural Sciences, Magyar Tudósok Körútja 2., H-1117 Budapest, Hungary

* Corresponding author: Tel: 36-1-463-4337, E-mail: pregi.emese@mail.bme.hu

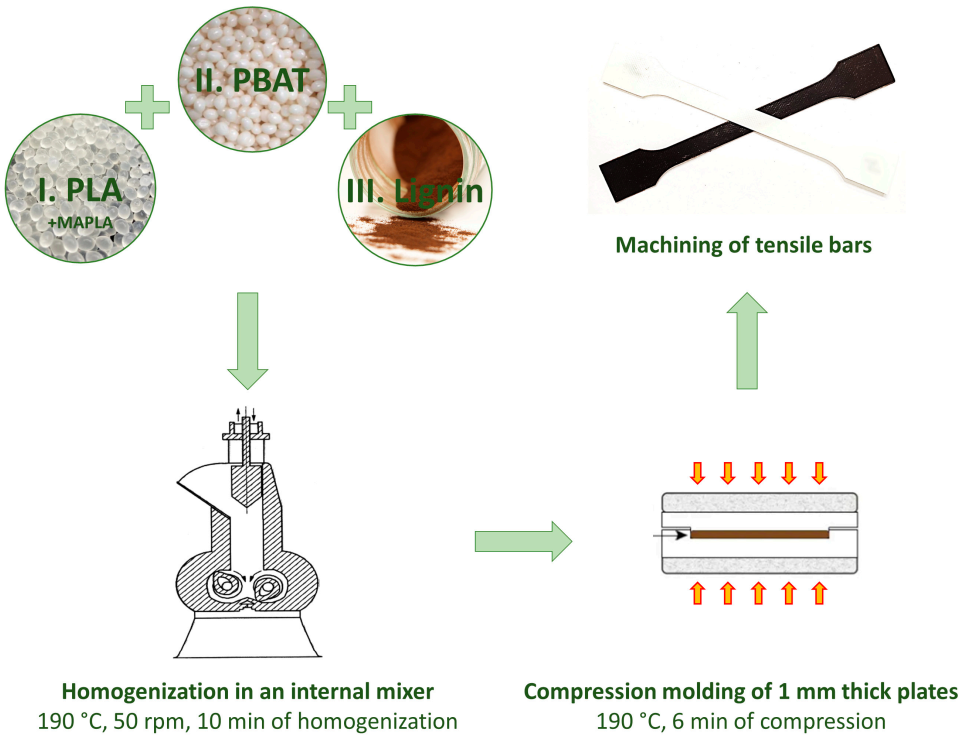

Figure S1. The preparation process of the samples

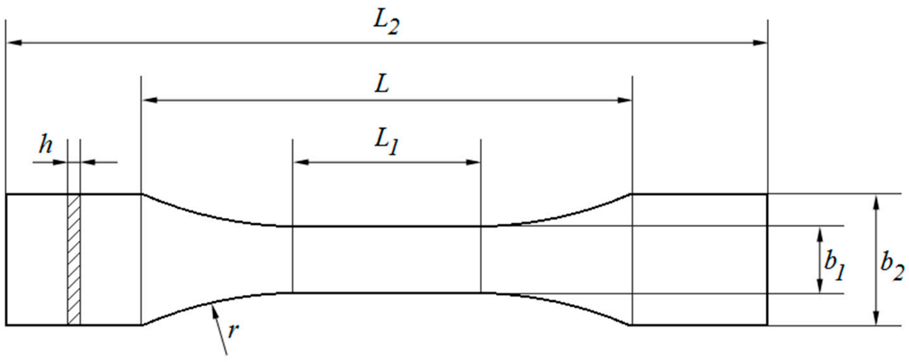

Figure S2. Shape and dimensions of tensile test specimens

Table S1 Dimensions of tensile test specimens

b_1	width at narrow portion	10 mm
b_2	width at ends	20 mm
h	thickness	1 mm
L_1	lenght of narrow parallel-sided portion	35 mm
L	initial distance between grips	75 mm
L_2	overall length	115 mm
r	radius	50 mm

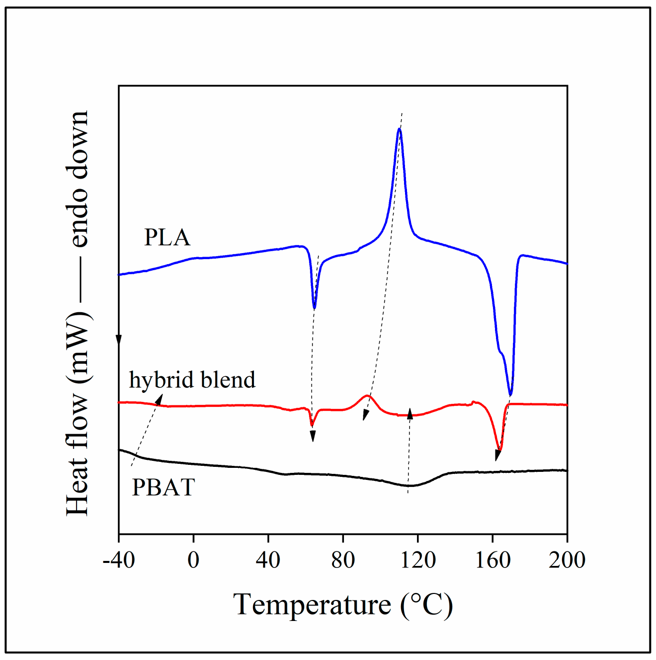

Figure S3. The result of a DSC measurement on a neat PLA, neat PBAT and PLA/lignin/PBAT blend containing 20 vol% lignin, 60 vol % PBAT and the functionalized PLA. First heating run.

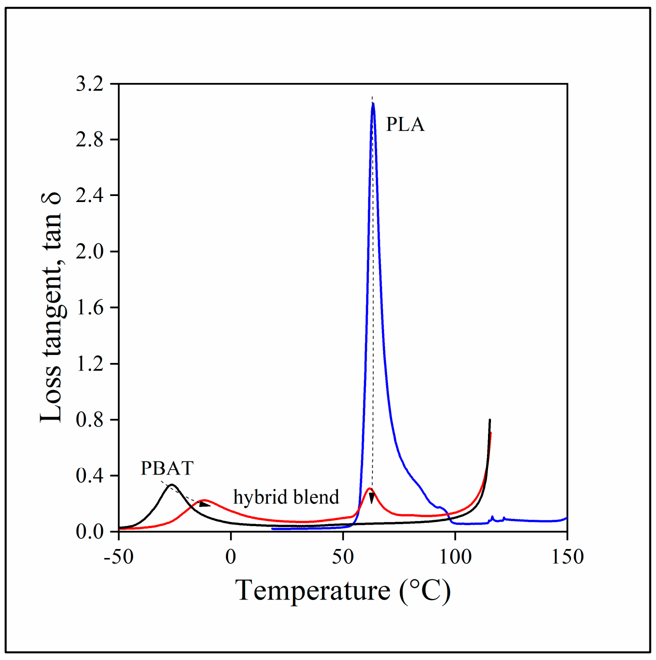

Figure S4. Dynamic mechanical spectra recorded on the neat PLA, neat PBAT and PLA/lignin/PBAT blend containing 20 vol% lignin, 60 vol% PBAT and MAPLA; temperature dependence of tan δ.

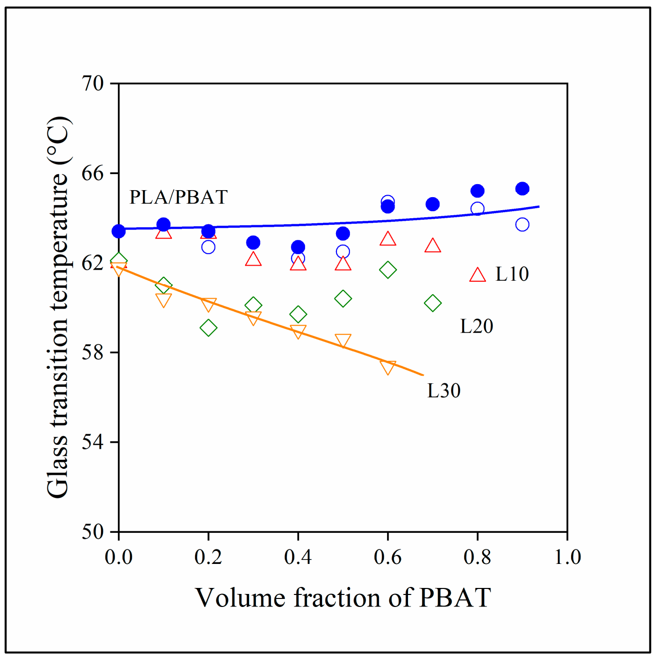

Figure S5. Composition dependence of the glass transition temperature of PLA in the two- and three-component blends studied. Symbols: (●) PLA/PBAT; (■) PBAT/lignin; lignin content in three-component blends: (△) 10, (◇) 20, (▽) 30 vol%. Empty symbols with and full symbols without MAPLA.

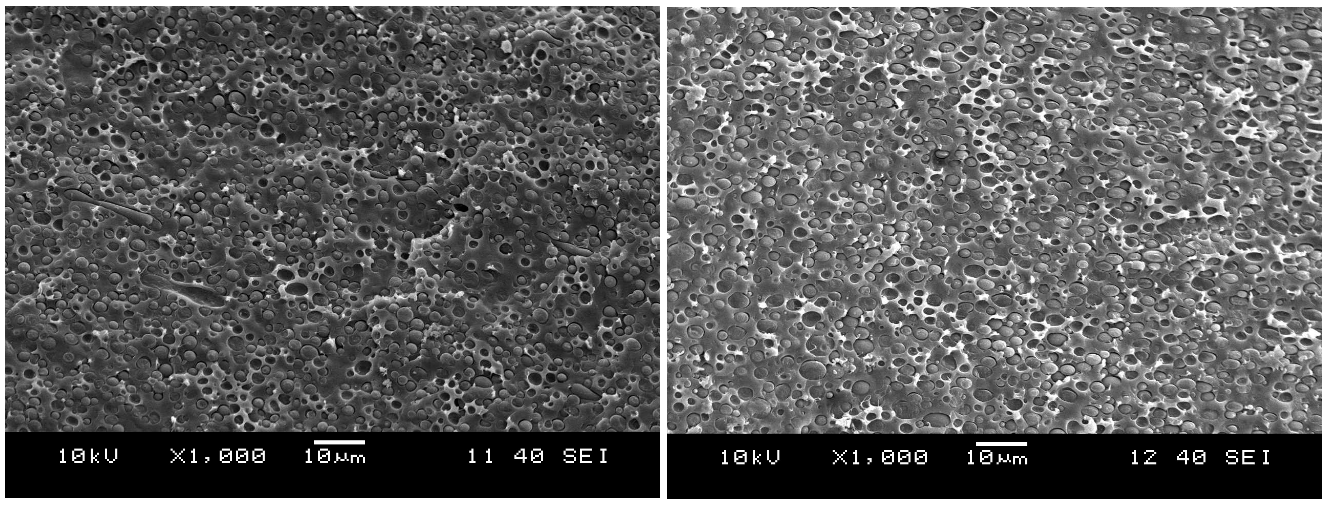

(**a**) (**b**)

Figure S6. Micrographs recorded on the structure of two-component PLA/PBAT blends containing 30 vol% of PBAT: (**a**) with MAPLA; (**b**) without MAPLA.

Table S2. Mechanical properties of two-component blends.

Sample	Modulus (GPa)		Tensile strength (MPa)		Elongation-at-break	
	average	std. dev.	average	std. dev.	average	std. dev.
PLA	3.32	0.091	55.89	1.246	2.98	0.253
PLA-L10	3.48	0.071	53.04	0.856	1.87	0.028
PLA-L20	3.50	0.135	32.04	2.258	0.61	0.090
PLA-L30	3.60	0.050	19.84	2.425	0.31	0.007
PLA-L40	3.74	0.014	9.94	0.919	2.98	0.253
PLA-PBAT10	2.91	0.032	25.42	8.153	22.4	6.49
PLA-PBAT20	2.51	0.056	30.42	2.201	79.0	19.07
PLA-PBAT30	1.93	0.091	25.33	1.067	128.4	59.85
PLA-PBAT40	1.37	0.023	18.24	1.338	100.2	12.13
PLA-PBAT50	0.78	0.064	16.54	0.661	28.5	11.84
PLA-PBAT60	0.35	0.019	12.15	0.761	50.7	11.87
PLA-PBAT70	0.21	0.006	12.32	0.220	180.0	18.85
PLA-PBAT80	0.16	0.004	15.83	0.724	430.3	33.05
PLA-PBAT90	0.11	0.006	17.03	0.537	781.9	30.15
PBAT	0.10	0.015	20.32	0.000	900.0	0.00
PBAT-L10	0.12	0.006	14.73	0.113	708.2	45.03
PBAT-L20	0.13	0.010	12.58	0.731	306.2	32.51
PBAT-L30	0.15	0.015	12.57	0.291	272.3	17.43
PBAT-L40	0.22	0.012	9.68	0.110	98.3	6.10
PBAT-L50	0.34	0.014	9.51	0.170	34.9	5.69
PBAT-L60	0.74	0.045	10.65	0.272	12.6	2.33

Table S3. Mechanical properties of two-component blends containing MAPLA.

Sample	Modulus (GPa)		Tensile strength (MPa)		Elongation-at-break	
	average	std. dev.	average	std. dev.	average	std. dev.
PLA	3.32	0.091	55.89	1.246	2.98	0.253
PLA-M-L10	3.52	0.095	44.53	4.455	1.43	0.148
PLA-M-L20	3.53	0.078	30.92	2.978	0.98	0.071
PLA-M-L30	3.52	0.344	11.43	0.419	0.35	0.036
PLA-M-L40	3.11	0.318	3.76	0.573	0.17	0.007
PLA- M-PBAT10	2.82	0.091	29.33	0.991	4.44	0.949
PLA- M-PBAT20	2.37	0.034	27.07	0.924	6.47	0.681
PLA- M-PBAT30	1.92	0.056	24.13	0.798	6.32	0.769
PLA- M-PBAT40	1.55	0.061	25.59	1.651	6.29	0.397
PLA- M-PBAT50	0.92	0.051	16.31	1.106	13.5	2.41
PLA- M-PBAT60	0.34	0.026	12.46	0.234	38.7	2.56
PLA- M-PBAT70	0.23	0.006	13.39	0.035	208.5	25.68
PLA- M-PBAT80	0.17	0.010	17.02	0.493	472.1	7.07
PLA- M-PBAT90	0.12	0.001	17.26	2.376	856.5	5.12
PBAT	0.10	0.015	20.32	0.000	900.0	0.00

Table S4. Mechanical properties of three-component hybrid blends containing MAPLA.

Sample	Modulus (GPa)		Tensile strength		Elongation-at-break	
	average	std. dev.	average	std. dev.	average	std. dev.
PLA-M-L10	3.52	0.095	44.53	4.455	1.43	0.148
PLA-M-L10-PBAT10	2.81	0.033	36.21	0.680	4.44	0.949
PLA-M-L10-PBAT20	2.24	0.028	27.35	1.905	6.47	0.681
PLA-M-L10-PBAT30	1.79	0.008	25.66	0.711	6.32	0.769
PLA-M-L10-PBAT40	1.28	0.015	20.49	0.528	6.29	0.397
PLA-M-L10-PBAT50	0.58	0.015	11.30	0.304	13.5	2.41
PLA-M-L10-PBAT60	0.28	0.008	10.00	0.218	38.7	2.56
PLA-M-L10-PBAT70	0.19	0.010	9.90	0.272	208.5	25.68
PLA-M-L10-PBAT80	0.14	0.017	13.02	0.636	472.1	7.07
PLA-M-L20	3.53	0.078	30.92	2.978	0.98	0.071
PLA-M-L20-PBAT10	2.49	0.061	25.75	0.729	1.34	0.073
PLA-M-L20-PBAT20	1.81	0.054	18.16	0.847	1.57	0.058
PLA-M-L20-PBAT30	1.35	0.022	12.82	0.606	1.86	0.137
PLA-M-L20-PBAT40	0.79	0.076	8.04	0.395	5.81	1.160
PLA-M-L20-PBAT50	0.32	0.011	9.12	0.163	17.9	3.07
PLA-M-L20-PBAT60	0.25	0.006	9.22	0.173	49.3	6.74
PLA-M-L20-PBAT70	0.17	0.012	10.44	0.025	281.2	20.84
PLA-M-L30	3.52	0.344	11.43	0.419	0.35	0.036
PLA-M-L30-PBAT10	2.64	0.038	21.99	1.079	1.00	0.061
PLA-M-L30-PBAT20	1.67	0.036	14.47	0.661	1.63	0.119
PLA-M-L30-PBAT30	1.11	0.035	7.50	0.169	2.07	0.252
PLA-M-L30-PBAT40	0.51	0.010	9.72	0.148	8.95	0.824
PLA-M-L30-PBAT50	0.32	0.015	8.52	0.312	27.7	3.45
PLA-M-L30-PBAT60	0.22	0.008	8.81	0.367	97.8	18.68
